# Supplementary material for: p27Kip1 Deficiency Impairs Brown Adipose Tissue Function Favouring Fat Accumulation in Mice
Source: Int J Mol Sci. 2023 Jan 31;24(3):2664. doi: 10.3390/ijms24032664 (PMC9916555; doi:10.3390/ijms24032664)
Supplement: Supplementary file 1 [file ijms-24-02664-s001.zip › ijms-2027336-supplementary.pdf]

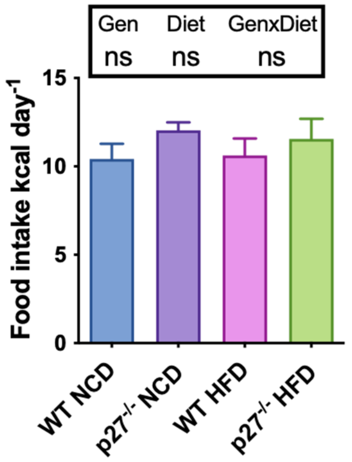

Supplemental Figure S1. Daily food intake in WT and p27$^{-/-}$ mice fed with NCD or HFD for 10 weeks. Legend above subfigures shows the result of the two-way ANOVA analysis; ns: non significant. Data are presented as mean ± SEM (n=4-7).

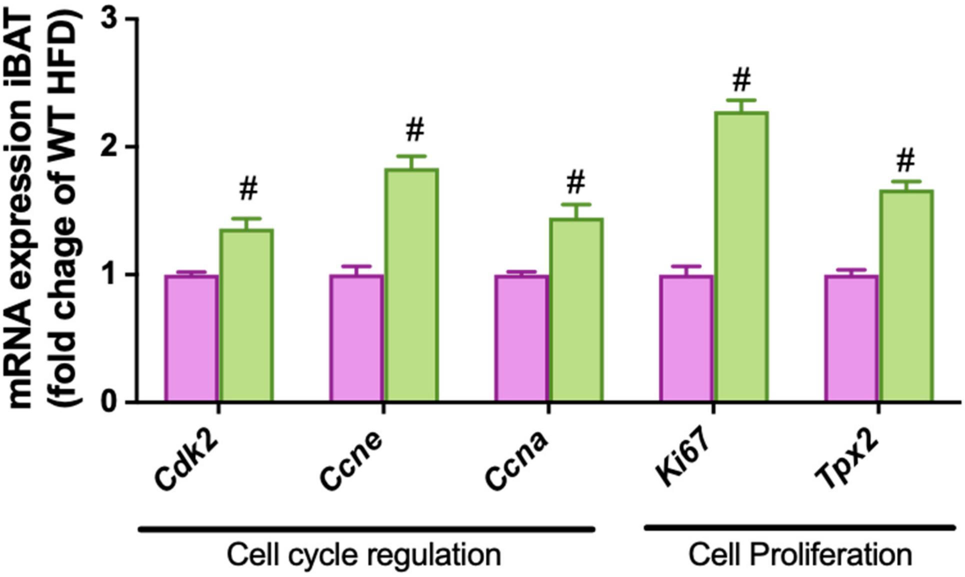

Supplemental Figure S2. mRNA expression analysis of genes involved in cell cycle regulation and in cell proliferation in iBAT of WT and *p27*$^{-/-}$ male mice fed with HFD for 10 weeks. Data are expressed as mean ± SEM (n=3-7). $^{\#}$p<0.05 *vs.* WT HFD.

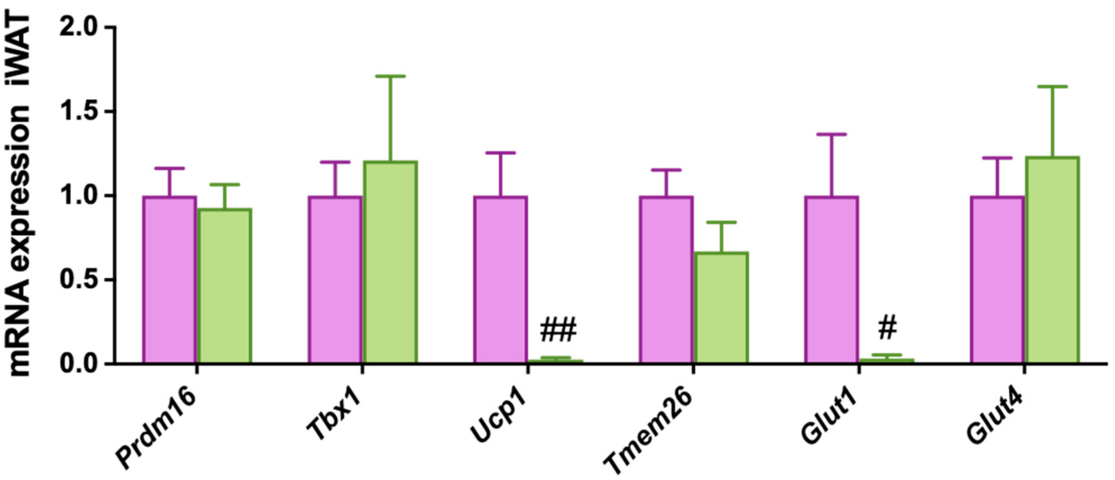

Supplemental Figure S3. **iWAT of *p27*$^{-/-}$ HFD mice have increased adipocyte size and decreased expression of *Ucp1* and *Glut1*. (a-b)** Representative histological images **(a)** and adipocyte area quantification **(b)** of the iWAT of WT and *p27*$^{-/-}$ mice fed with HFD for 10 weeks. **(c)** mRNA expression levels of relevant genes for adipose tissue browning and glucose uptake in WT and *p27*$^{-/-}$ mice fed with HFD for 10 weeks. Data are expressed as mean ± SEM (n=5-9). $^{\#}$p<0.05, $^{\#\#}$p<0.01, $^{\#\#\#}$p<0.001 vs. WT HFD.

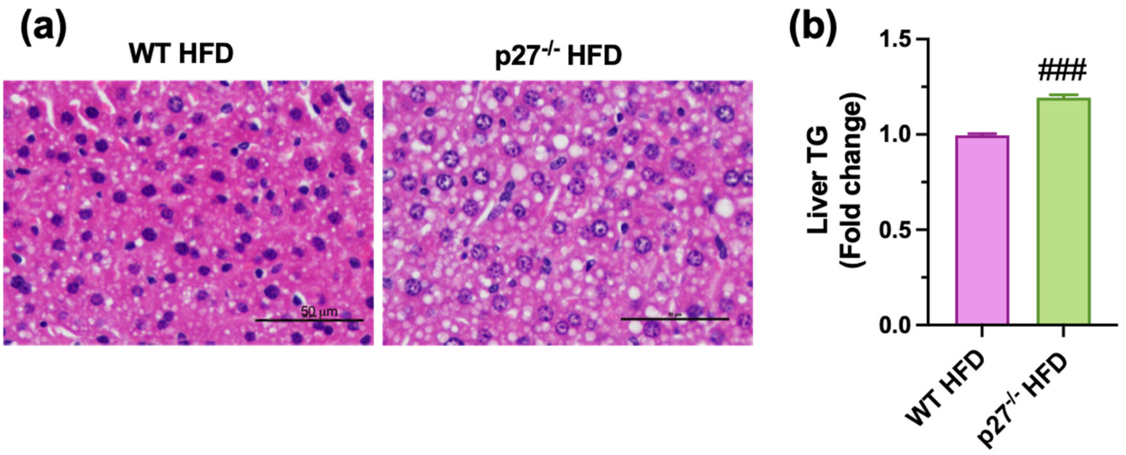

Supplemental Figure S4. Representative histological images **(a)** and hepatic triglyceride content **(b)** of the liver of WT and *p27*$^{-/-}$ mice fed with HFD for 10 weeks. Bar = 50 um. $^{\#\#\#}$p<0.001 vs. WT HFD. (n=7-9)
